# Coronatine Gene Expression *In Vitro* and *In Planta*, and Protein Accumulation During Temperature Downshift in *Pseudomonas syringae*

**DOI:** 10.3390/s90604272

**Published:** 2009-06-03

**Authors:** Yvonne Braun, Angela V. Smirnova, Helge Weingart, Alexander Schenk, Matthias S. Ullrich

**Affiliations:** 1 Jacobs University Bremen, School of Engineering and Science, Campus Ring 1, 28759 Bremen, Germany; E-Mails: angelasmirnova@hotmail.com (A.V.S.); h.weingart@jacobs-university.de (H.W.); a.schenk@jacobs-university.de (A.S.); m.ullrich@jacobs-university.de (M.S.U.); 2 National Institute for Medical Research, The Ridgeway, London NW7 1AA, UK

**Keywords:** COR, coronatine, TCS, two-component system, HPK, histidine protein kinase, RR, response regulator

## Abstract

The plant pathogenic bacterium *Pseudomonas syringae* PG4180 synthesizes high levels of the phytotoxin coronatine (COR) at the virulence-promoting temperature of 18 °C, but negligible amounts at 28 °C. Temperature-dependent COR gene expression is regulated by a modified two-component system, consisting of a response regulator, CorR, the histidine protein kinase CorS, and a third component, termed CorP. We analyzed at transcriptional and translational levels the expression of *corS* and the *cma* operon involved in COR biosynthesis after a temperature downshift from 28 to 18 °C. Expression of *cma* was induced within 20 min and increased steadily whereas *corS* expression was only slightly temperature-dependent. Accumulation of CmaB correlated with accumulation of *cma* mRNA. However, *cma* transcription was suppressed by inhibition of *de novo* protein biosynthesis. A transcriptional fusion of the *cma* promoter to a promoterless *egfp* gene was used to monitor the *cma* expression *in vitro* and *in planta*. A steady induction of *cma::egfp* by temperature downshift was observed in both environments. The results indicate that PG4180 responds to a temperature decrease with COR gene expression. However, COR gene expression and protein biosynthesis increased steadily, possibly reflecting adaptation to long-term rather than rapid temperature changes.

## 1. Introduction

In order to survive, bacteria constantly have to adapt to changing environmental conditions. Environmental stimuli can serve pathogenic bacteria as signals for expression of virulence factors, for example, temperature often serves as a trigger for the induction of virulence gene expression. While temperatures of warm-blooded hosts (37-41 °C) are important signals for the induction of virulence gene expression in humans and animal pathogens [1], plant pathogens generally express their virulence genes in response to lower temperatures [2-4]. Further, the later organisms adjust their virulence gene expression in response to diurnal and seasonal temperature fluctuations.

COR is a non-host specific phytotoxin, which is an important virulence factor of several pathovars of *Pseudomonas syringae*, precisely pv. *alisalensis, atropurpurea, glycinea, maculicola, morsprunorum, porri*, and *tomato*. Biosynthesis of COR is positively regulated by low temperature in *P. syringae* pv. *glycinea* PG4180 [4, 5]. PG4180 synthesizes COR in a temperature-dependent manner, with a maximum at 18 °C [5]. At 28 °C, the optimal growth temperature of *P. syringae*, COR biosynthesis is negligible. COR consists of the polyketide coronafacic acid (CFA) and the cyclised amino acid coronamic acid (CMA), an isoleucin derivative. CFA and CMA are linked by the formation of an amide bond. The genes required for CFA and CMA biosynthesis are organized as two biosynthetic operons, *cfl*/CFA and CMA, respectively [6,7]. Nucleotide sequence analysis of the CMA biosynthetic genes has suggested that this precursor is synthesized by a mechanism similar to the one used in the biosynthesis of non-ribosomal peptides [7-9]. In the CMA cluster seven genes have been identified. Six of them, *cmaABCDET*, encode the enzymes for biosynthesis of CMA from the progenitor L-*allo*-isoleucine [10]. Until today no function has been assigned to the seventh gene, *cmaU*. CFA is a polyketide and its synthesis resembles the biosynthesis of polyketides in *Streptomyces* and *Bacillus* spp. [11]. The CFA biosynthetic cluster contains nine genes. Two encode type I polyketide synthase proteins (*cfa6* and *cfa7*), the genes *cfa1, cfa2*, and *cfa3* encode proteins similar to the acyl carrier protein, dehydratase, and beta-ketoacyl:acyl carrier protein synthase of type II fatty acid synthases and polyketide synthases [12].

One means to sense and respond to environmental stimuli are two-component regulatory systems (TCSs). A modified TCS consisting of a histidine protein kinase (HPK), CorS, a response regulator (RR), CorR, and a third component, CorP, regulates COR production at the transcriptional level in *P. syringae* [5]. CorP showed high similarity to CorR but lacks a DNA binding domain characteristic for RRs. Mutational analysis demonstrated that CorP is necessary for induction of COR biosynthesis [5], but its exact function remained to be determined. The HPK, CorS, is believed to respond to a temperature change via autophosphorylation of a conserved histidine residue, and transduces the signal to the cognate RR CorR via phosphorylation of its conserved aspartate residue [13]. *In vitro* results indicated that CorR was able to bind to the CMA and CFA biosynthetic promoter regions in a temperature- and *corS*-dependent manner [14,15]. Additionally, the alternative sigma factor RpoN (σ^54^) which is required for the expression of a variety of virulence determinants and metabolic functions was shown to be essential for COR biosynthesis in *P. syringae* [16]. Consequently, COR gene expression seems to be regulated by its specific TCS and by at least one global regulator.

In natural settings PG4180 encounters temperature fluctuations and adapts COR gene expression accordingly. In this study, we investigated effects of a temperature shift from 28 to 18 °C on transcription of CMA biosynthetic genes *in vitro* as well as *in planta* and on CmaB protein biosynthesis. We also evaluated transcription of the regulatory gene *corS* after the temperature shift and studied whether *de novo* protein biosynthesis is required for transcriptional activation of COR biosynthetic genes. Taking into account that stability of mRNA contributes to mRNA levels and that mRNA stability is also influenced by temperature, we investigated the stability of the *cma* transcript at 18 and 28 °C after inhibition of transcriptional initiation. The HPK CorS is a membrane-associated protein, which possesses a hydrophobic *N*-terminus comprising six transmembrane domains (TMDs) [17]. HPKs of this structure are generally believed to sense environmental stimuli by means of their periplasmic loops. In some sensory proteins periplasmic domains for substrate binding were identified, e.g. the nitrate binding Nit domain of the sensor histidine kinases NarX and NarQ of *E. coli* [18]. Another example is the citrate binding CitAP domain in the CitA sensor kinase of *Klebsiella pneumoniae* [19]. In the case of CorS, a temperature decrease results in COR biosynthesis. Whether the temperature change on its own or an additional signal activates the regulatory cascade remains to be elucidated.

## 2. Materials and Methods

### 2.1. Bacterial strains, plasmids, and growth conditions

*P. syringae* pv. glycinea PG4180 [20] was maintained at 28 °C on MG agar plates [21]. For liquid cultures bacteria were grown at 18 and 28 °C with constant shaking at 280 rpm in HSC medium [4]. For comparison of the *cma* expression *in vitro* and *in planta*, PG4180 carrying plasmid pHW01 was used [22]. Plasmid pHW01 contains a transcriptional fusion of a promoterless enhanced green fluorescent protein (*egfp*) gene to the *cma* promoter region of PG4180 in the broad-host range vector pBBR1MCS [23]. The size of the cloned *cma* promoter region is 2.9 kb located at positions -717 to +2,135 with respect to the *cmaA* transcriptional start site [7].

### 2.2. Plant material and inoculation procedures

Soybean plants (*Glycine max* (L.) Merr. cv. Maple Arrow) were grown in a greenhouse at 22 to 25 °C, 60% humidity, with supplementary light for a 14-h photoperiod (350 μE m^-2^ s^-1^). To study expression of the *cma* promoter *in planta, P. syringae* PG4180 carrying plasmid pHW01 was infiltrated into soybean leaves using a needleless syringe at an OD_600_ of 0·05 (approx. 5 × 10^7^ CFU per mL). Following infiltration, plants were transferred to growth chambers at 18 or 28 °C. To recover bacteria after inoculation, 60 discs (7 mm diameter) containing infected leaf tissue were excised using a cork borer and macerated in 4 mL of isotonic NaCl. All greenhouse experiments were repeated at least three times to confirm reproducibility.

### 2.3. Confocal laser scanning microscopy

Fluorescence of bacterial cells was detected with a confocal laser scanning microscope, DMR XE, type TCS NT (Leica) using a PL-Fluotar objective (× 63; 1.32; numerical aperture, oil immersion). Images were analyzed with Leica software package TCS NT, version 1.5.451.

### 2.4. RNA isolation and spot blot analysis

Bacteria were cultured in HSC medium at 18 and 28 °C until OD_600_ of 1·3 or until selected time points after temperature shift or addition of antibiotics. 15 mL of bacterial culture were mixed with an equal volume of chilled killing buffer (20 mM Tris-HCl [pH 7.5], 20 mM NaN_3_) and subsequently centrifuged at 4 °C for 15 min at 4,000 rpm.

Total RNA was isolated from 5 mL of bacterial cells by acid phenol/chloroform extraction as described by Schenk *et al.* [24]. Extracted RNA was analysed using an Agilent 2,100 Bioanalyzer. The yield of RNA was determined spectrophotometrically at 260 nm using an Ultrospec 2,100 pro UV/Visible Spectrophotometer (Amersham). Aliquots of total RNA (200 ng per dot) were transferred to positively charged nylon membranes (Pall) using the Minifold^®^ I Spot-Blot System (Schleicher & Schuell BioScience) according to the manufacturer's recommendations. Successful transfer of the RNA was verified by reversible staining of the membrane with methylene blue [25]. The digoxygenin-labelled specific RNA probes were synthesized by *in vitro* transcription using T7 RNA polymerase and specific PCR products as templates. Synthesis of the templates by PCR was performed using the following pairs of oligonucleotides: for the *cmaA* probe, primers cmaA-fwd (5′-TTTGAGTCGGTCTGCACGCA-3′) and cmaA-revT7 (5′-TAATACGACTCACTATAGGGAGGGCTGTACGTTGTCTACTAG-3′), for *corS*, corS-fwd (5′-AATACGGCGCGCTGTCAGTT-3′) and corS-revT7 (5′-TAATACGACTCACTATAGGGAGGAATGGAT GGCCTAATAGGCG-3′) for *ssb*, ssb-fwd (5′-ATGGCCCGTGGGGTTAACAAAGTC-3′) and ssb-revT7 (5′-TAATACGACTACTATAGGGAGGTGTCAAAGTCAGCAGCAGGC-3′). For the synthesis of DIG-labelled RNA probes, the Strip-EZ™ RNA Probe Synthesis & Removal kit (Ambion) and digoxygenin-11-UTP (Roche) were used. Before hybridization with a probe, RNA was cross-linked with the membrane under a UV-light using a CL-1000 Crosslinker (UVP). After 1 h of pre-hybridization at 68 °C the probe was hybridized to the membrane by incubation in hybridization solution (50% (v/v) formamide, 7% (w/v) SDS, 2% (w/v) blocking reagent, 0.1% (v/v) *N*-laurylsarcosine, 5 × SSC) at 68 °C for 12 – 16 h. After hybridization, the membrane was washed twice for 5 min at room temperature in 2 × SSC containing 0.1% (w/v) SDS, followed by two washes for 15 min at 68 °C in 0.2 × SSC containing 0.1% (w/v) SDS. Hybridization signals were detected by incubation with anti-digoxygenin-AP Fab fragments (Roche) and a fluorescence substrate for alkaline phosphatase (ECF) (Amersham-Pharmacia Biotech) using a FLA-3000 phosphoimager (Raytest, Strabenhardt, Germany). Signals were quantified using the AIDA Image Analyzer software package (Raytest). The specificity of the RNA probes was evaluated by Northern Blot analysis (data not shown).

### 2.5. Immunodetection of CmaB accumulation

Total protein extracts were isolated from cell pellets of bacterial cultures (1.5 mL) and were quantified by the Bradford assay [26]. Equal amounts of protein (30 μg per lane) were separated by 10%-SDS-PAGE on two gels running in the same chamber. Subsequently, proteins from one gel were electrotransferred to a Hybond-C nitrocellulose membrane (Amersham-Pharmacia Biotech). The second gel was stained with GelCode Blue Stain Reagent (Perbio Science). Polyclonal mouse antiserum raised against CmaB (dilution 1:1,000) was used to monitor the accumulation of CmaB after a temperature shift from 28 to 18 °C. Secondary anti-mouse IgG antibodies conjugated to alkaline phosphatase (Sigma) at 1:10,000 dilution, along with ECF (Amersham-Pharmacia Biotech), a fluorescence substrate for alkaline phosphatase, were used for detection of signals. Fluorescence was detected using a FLA-3000 phosphoimager (Raytest). Signals were quantified using the AIDA Image Analyzer software package (Raytest).

## 3. Results and Discussion

The plant pathogen *P. syringae* PG4180 often encounters temperature fluctuations during its epiphytic growth on plant surfaces. Besides diurnal changes, temperature decreases are often associated with rainy or humid weather conditions, which favour bacterial infection of the host plant (soybean) and affect the subsequent disease development [27,28]. In PG4180, a low-temperature stimulus is known to be sensed and to cause coordinated COR gene expression via the CorRSP regulatory system [5]. However, important questions that remained to be addressed were how the temperature stimulus is transmitted to COR gene expression and how rapidly the COR gene expression is induced *in vitro* as well as *in planta* when temperature changes. In this study, a significant induction of the *cma* biosynthetic operon and an increased synthesis of CmaB following a temperature downshift from 28 to 18 °C were demonstrated.

### 3.1. Analysis of cma and corS mRNA levels upon a temperature shift from 28 to 18 °C

**Figure 1.**
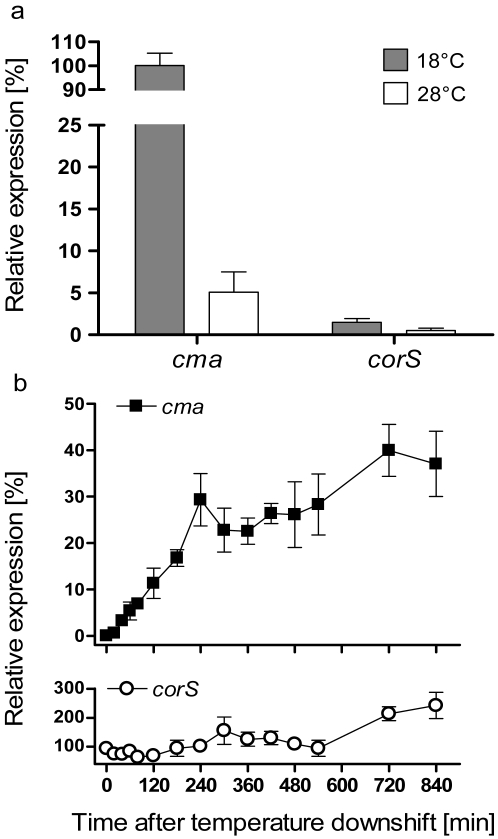
Synthesis of *cma* and *corS* mRNAs at 18 °C, 28 °C and after a temperature downshift from 28 to 18 °C, as determined by RNA spot blot analysis. (A) RNA spot blot data for *cma* and *corS* mRNAs at 18 and 28 °C. Samples were withdrawn from cultures at OD_600_ of 1·0. mRNA levels at 18°C were defined as 100%. (B) RNA spot blot analysis for *cma* and *corS* mRNAs after a temperature downshift from 28 to 18°C. Bacteria were grown in HSC medium at 28 °C to an OD_600_ of 0·5 and subsequently shifted to 18 °C. Total RNA was isolated from samples withdrawn from PG4180-cultures at different time points after the downshift in temperature and subjected to RNA spot blot analysis. Quantities represent averages of two experiments with six replicates. The relative mRNA level is related to the level of the respective mRNA synthesized at 18 °C (A), which was defined as 100%.

In order to investigate how rapidly the induction of transcription of COR biosynthetic genes occurs after a temperature change, levels of *cma* and *corS* mRNA after a temperature shift from 28 to 18 °C were examined by RNA spot blot analysis. PG4180 cultures were grown in HSC medium at 28 °C to an OD_600_ of 0·5. Subsequently, one set of aliquots was shifted to 18 °C. Cultures continuously kept at 28 °C were used as a control to define the minimal level of expression. Additionally, cultures grown continuously at 18 °C were used as a control to define the maximal level of expression. Determination of the minimal and maximal levels, respectively, showed that the level of *cma* transcript was 20-fold higher at 18 than at 28 °C (Figure 1a).

However, temperature induction of the *corS* transcript was only 4-fold (Figure 1a) indicating that *corS* expression was only moderately affected by temperature in comparison to *cma*. Upon downshift in temperature, a slight increase of mRNA levels for the *cma* transcript occurred within 20 min (Figure 1b). Subsequently, the *cma* transcript accumulated gradually and reached a maximum concentration after 12 hours of growth at 18 °C. During the following hours *cma* mRNA remained at this level with minor fluctuations. 24 hours after the downshift the expression of *cma* had not reached levels measured in cultures constantly grown at 18 °C. The levels of mRNA in the 28 °C cultures remained at basal level (data not shown). The level of *corS* mRNA increased transiently upon temperature-downshift. 14 h after the temperature downshift a 2.5-fold increase of this transcript was determined and remained constant for the analyzed period of 24 h. Thus, transcription of the regulatory gene *corS* was not markedly affected by temperature. This is not surprising since regulatory genes such as *corS* need to be transcribed constitutively to allow their gene products to quickly react to environmental changes.

It had previously been reported that low temperature induces synthesis of cold-shock proteins [29] and desaturases in *Bacillus* and *Synechocystis* species, respectively [30,31]. Expression studies for the *des* gene encoding Δ5 desaturase of *B. subtilis* demonstrated a transient increase in *des* mRNA levels in response to a sudden change in temperature. The levels of the *des* transcript reached a maximum within 60 min and subsequently decreased after continuous growth at low temperature [30]. The synthesis of cold-shock proteins is also transiently induced and then re-adjusted to new steady-state levels within 4 h of a temperature downshift [32]. In contrast, the *cma* mRNA of PG4180 accumulated steadily and gradually after the shift from 28 to 18 °C over a period of 24 h (Figure 1b, data shown up to 14 h) indicating that it does not resemble a typical cold shock response.

**Figure 2.**
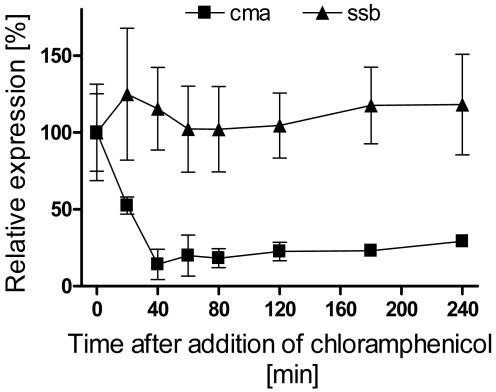
Synthesis of *cma* and *ssb* mRNAs following a temperature downshift from 28 to 18 °C and addition of chloramphenicol (100 μg mL^-1^). Total RNA was isolated from samples withdrawn from PG4180 cultures at different time points and subjected to RNA spot blot analysis using *cma*- and *ssb*-specific probes. The amount of detected mRNA at zero minutes was considered as 100%. Quantitative data represent averages of two experiments with six replicates.

To examine whether or not the induction of the *cma* transcription in PG4180 cultures shifted from 28 to 18 °C required *de novo* protein synthesis, levels of *cma* mRNA were measured after addition of the protein biosynthesis inhibitor, chloramphenicol (100 μg mL^-1^). After addition of chloramphenicol the level of *cma* mRNA decreased in the shifted culture (Figure 2). In parallel, samples were also probed for the *ssb* transcript encoding single-strand binding protein. The levels of *ssb* mRNA were not affected by addition of chloramphenicol. Thus, inhibition of *de novo* protein synthesis suppressed transcription of *cma* but not of *ssb*.

**Figure 3.**
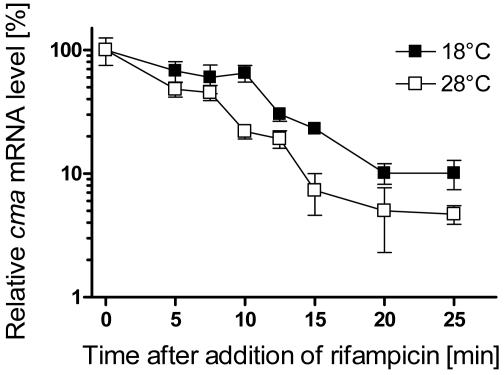
Stability of the *cma* transcript as a function of temperature. Transcription was blocked by addition of rifampicin (0 min) at 50 μg mL^-1^ to the 18 °C-culture and the culture shifted from 18 to 28 °C at an OD_600_ of 1·0. Total RNA was isolated from aliquots harvested at different time points after rifampicin addition and subjected to RNA spot blot analysis. The amount of detected mRNA at zero minutes was considered as 100%. The percentage of transcript remaining after rifampicin addition was plotted as a function of time.

To determine whether or not a change in mRNA stability contributed to the temperature-induced increase in mRNA levels, the decay of the *cma* mRNA was monitored in cultures of PG4180 grown at 18 °C or shifted from 18 to 28 °C after addition of rifampicin. This antibiotic inhibits initiation of transcription. Rifampicin was added at 50 μg mL^-1^ to cultures of PG4180 at OD_600_ of 1·0. As seen from Figure 3, the *cmaA* transcript was degraded faster at 28 as compared to 18 °C.

The half-life of *cma* mRNA was 9.9 min at 18 °C and 7.3 min at 28 °C as calculated by linear regression. This result indicated that the stability of the c*ma* transcript was rather moderately affected by a temperature change. Transcripts are generally more stable at lower temperatures. The slightly higher stability of the *cma* transcript at 18 compared to 28 °C seems to be due to normal slower degradation. Therefore, the increase in *cma* transcript at 18°C is rather a effect of accelerated transcription than of increased stability.

### 3.2. Analysis of CmaB protein levels following a temperature shift from 28 to 18 °C

**Figure 4.**
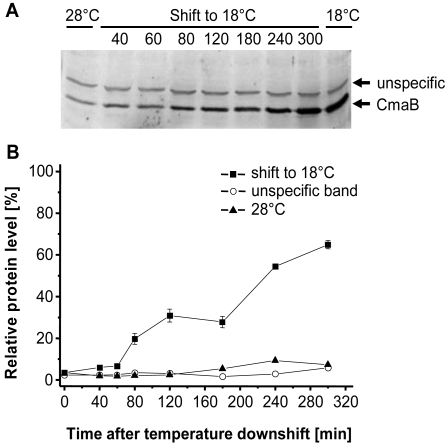
Effect of temperature downshift on the levels of CmaB. Quantitative Western blot analysis of cells shifted from 28 to 18 °C using polyclonal mouse antiserum against CmaB. The time points of sampling are indicated in minutes. The levels of CmaB and of an unknown protein that immunoreacted nonspecifically with the CmaB antiserum were quantified. The relative protein level is related to the level of CmaB synthesized at 18 °C, which was defined as 100%. Quantitative data represent averages of two experiments with two replicates.

The genes in the *cma* operon encode enzymes which resemble non-ribosomal peptide synthetases [7]. CmaB belongs to a new class of O_2_-dependent non-heme iron halogenases [36] that catalyzes a chlorination reaction of the CMA precursor [9]. To monitor CmaB production after the temperature downshift, 1.5 mL samples were drawn from PG4180 cultures as previously done for RNA spot blot experiments. Protein extracts were subjected to Western blot analysis using a CmaB polyclonal antiserum (Figure 4). In the first five hours after the temperature downshift, levels of CmaB increased gradually and reached 65% of the CmaB levels detected in cultures continuously grown at 18 °C (Figure 4). Generally, the accumulation of CmaB was consistent with the increase of *cma* mRNA levels. As controls, the level of CmaB was determined in cultures constantly grown at 28 °C at optical densities corresponding to the optical densities of the shifted culture. The amount of CmaB detected in this cultures remained always on a basal level (Figure 4). The highest level of CmaB, detected 5 h after the temperature downshift, was 9-fold higher in the shifted culture as compared to the 28 °C control. As shown in Figure 4, the CmaB antiserum also immuno-reacted non-specifically resulting in unspecific signals. However, intensities of the unspecific signals did not increase significantly, thus confirming a specific temperature-dependent accumulation of CmaB.

The steady accumulation of CmaB over the investigated period of 5 h together with the obtained mRNA accumulation (Figure 1) suggests that the effect of temperature downshift does not resemble a cold-shock response in *P. syringae*. These results were consistent with previously observed effects of temperature on other temperature-responsive genetic loci in PG4180 [33].

### 3.3. Analysis of cma promoter activity in planta following a temperature shift from 28 to 18 °C

In order to test whether temperature dependence of *cma* expression can also be seen in bacterial cells infecting host plants, the effects of a temperature downshift on *in planta* expression of *cma* were analyzed using cells of PG4180 (pHW01) isolated from soybean leaves. Plasmid pHW01 [22] allowed fluorescence-based monitoring of EGFP, a red-shifted variant of green fluorescent protein expressed as a transcriptional fusion of a promoterless *egfp* to the *cma* promoter region of PG4180. Soybean plants were inoculated with PG4180 (pHW01) grown at 28 °C, and subsequently kept in growth chambers at 18 °C. Bacteria were recovered from soybean leaves 1, 6, 12 and 24 hours after the inoculation. As *in vitro* control, a PG4180 (pHW01) culture grown in HSC medium at 28 °C was shifted to 18 °C at an OD_600_ of 0·5 and kept growing in HSC medium. Cells were harvested from the shifted culture after 1, 6, 12 and 24 hours of growth, resuspended in phosphate-buffered saline (pH 7.4) and adjusted to an OD_600_ of 0·5. EGFP fluorescence of bacteria grown *in vitro* and *in planta* was determined by confocal laser scanning microscopy. Fluorescence of cells constantly grown at 28 °C as control remained at a very low level throughout the entire growth of the bacteria *in vitro* and *in planta* [22].

**Figure 5.**
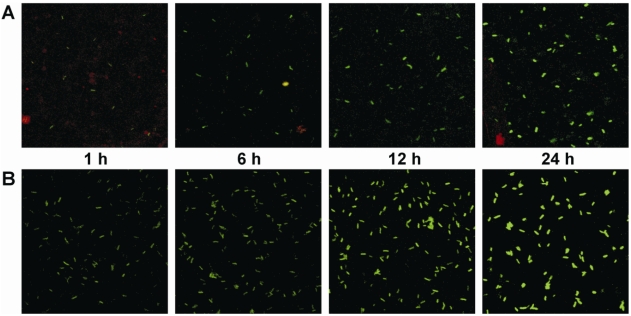
Fluorescence of *P. syringae* PG4180 (pHW01) after a temperature downshift *in planta* and *in vitro* imaged by confocal laser scanning microscopy. (A) Fluorescence of *P. syringae* PG4180 (pHW01) after a temperature downshift *in planta*. Plants were inoculated with bacteria grown at 28°C and subsequently kept in growth chambers at 18 °C. (B) Fluorescence of *P. syringae* PG4180 (pHW01) after a temperature downshift *in vitro*. Bacteria were grown in HSC medium at 28 °C to an OD_600_ of 0·5 and subsequently shifted to 18 °C. Cells were harvested at the indicated time points.

After 1 h, fluorescence signals of the cells were barely detectable *in planta* (Figure 5a). A slight increase in the fluorescence intensity occurred after 6 h of growth *in planta* at 18 °C. Fluorescence signals were significantly enhanced after 12 h, and a maximal increase of cell fluorescence was detected after 24 hours of growth *in planta* at 18 °C. Similar enhancement of fluorescence was observed for cells grown in HSC medium after the temperature downshift (Figure 5b). These results clearly demonstrated that the temperature effects observed *in vitro* were also relevant for PG4180 cells grown under *in planta* conditions. However, the intensity of the transcriptional signal as visualized with *egfp* from 12 to 24 h *in planta* seemed to be less pronounced *in vitro* when mRNA was quantified. One potential reason for this could be the intrinsic stability of EGFP. It had previously been shown that GFP has a half-life of more than 1 day *in vivo* [34]. Thus, the protein may accumulate in bacterial cells and be present even after the respective promoter became less active. Alternatively, the stability of the mRNA might have been influenced by the transcriptional fusion. Moreover, the promoter probing construct includes the *N*-terminus of the amino acid coding region of CmaA, therefore it can be considered as a CmaA:EGFP protein fusion as well. This might also be responsible for the altered signal levels. Additionally, it cannot be ruled out that plant-derived signals may have contributed to the stronger induction of COR gene expression.

## 4. Conclusions

The plant pathogen *P. syringae* PG4180 produces COR, an important virulence factor, in a temperature-dependent manner with a maximum at 18 °C. Temperature fluctuations occur constantly on the plant surface. Thus, it is likely that the plant pathogen PG4180 is adapted to alter its gene expression in response to an average (low or high) daily temperature. A decrease in the average temperature favours a steady increase of COR gene expression. COR is a secondary metabolite and therefore not essential for bacterial survival at low temperatures [8]. By this adaptation to long-term temperature decreases the bacteria may presumably induce the energy-consuming COR biosynthesis only under conditions which favour the infection of the host plant. COR is synthesized from two precursor molecules, CFA and CMA. Genes for CFA and CMA biosynthesis are encoded in two distinct operons, separated by three regulatory genes, corR, corS, and corP. The present study demonstrated a significant increase in *cma* expression in response to a temperature downshift *in vitro* and *in planta*. Additionally, accumulation of CmaB protein was observed after temperature downshift. Taken together, the results demonstrate that COR gene expression in PG4180 was efficiently activated by temperature downshifts *in vitro* as well as *in planta*. On the contrary, expression of the regulatory gene *corS* was only moderately affected by temperature change. Budde *et al.* [8] previously reported that the *cma* transcript was not detected in a *corR corS* mutant of PG4180. We suggest that the induction of *cma* transcription was governed by a “switch” in the signal transduction pathway performed by CorRSP in response to the temperature downshift.

At low temperature CorS might undergo a conformational change to an active form in order to be efficiently autophosphorylated and to subsequently interact with its cognate RR, CorR, to allow initiation of transcription of COR biosynthetic genes [17,35]. Inhibition of *de novo* protein synthesis prior to the temperature downshift resulted in a subsequent decrease of *cma* transcription. This result supports the hypothesis that activation of *cma* transcription requires *de novo* synthesis and membrane incorporation of an altered form of CorS. Alternatively, suppressed synthesis of RpoN after inhibition of *de novo* biosynthesis might affect *cma* transcription indirectly via CorR or another σ^54^-dependent activator [16]. In both cases, COR gene expression seems to be dependent on *de novo* biosynthesis of regulatory proteins after a temperature downshift.

## References

[1] Eriksson S., Hurme R., Rhen M. (2002). Low-temperature sensors in bacteria. Phil. Trans. R. Soc. Lond. B.

[2] Hugouvieux-Cotte-Pattat N., Dominguez H., Robert-Baudouy J. (1992). Environmental conditions affect transcription of the pectinase genes of *Erwinia chrysanthemi* 3937. J. Bacteriol..

[3] Jin S., Song Y.N., Deng W.Y., Gordon M.P., Nester E.W. (1993). The regulatory VirA protein of *Agrobacterium tumefaciens* does not function at elevated temperatures. J. Bacteriol..

[4] Palmer D.A., Bender C.L. (1993). Effects of environmental and nutritional factors on production of the polyketide phytotoxin coronatine by *Pseudomonas syringae* pv. glycinea. Appl. Environ. Microbiol.

[5] Ullrich M., Peñaloza-Vázquez A., Bailey A.M., Bender C.L. (1995). A modified two-component regulatory system is involved in temperature-dependent biosynthesis of the *Pseudomonas syringae* phytotoxin coronatine. J. Bacteriol..

[6] Liyanage H., Palmer D.A., Ullrich M., Bender C.L. (1995). Characterization and transcriptional analysis of the gene cluster for coronafacic acid, the polyketide component of the phytotoxin coronatine. Appl. Environ. Microbiol..

[7] Ullrich M., Bender C.L. (1994). The biosynthetic gene cluster for coronamic acid, an ethylcyclopropyl amino acid, contains genes homologous to amino acid-activating enzymes and thioesterases. J. Bacteriol..

[8] Budde I.P., Rohde B.H., Bender C.L., Ullrich M.S. (1998). Growth phase and temperature influence promoter activity, transcript abundance, and protein stability during biosynthesis of the *Pseudomonas syringae* phytotoxin coronatine. J. Bacteriol..

[9] Couch R., O'Connor S.E., Seidle H., Walsh C.T., Parry R. (2004). Characterization of CmaA, an adenylation-thiolation didomain enzyme involved in the biosynthesis of coronatine. J. Bacteriol..

[10] Kelly W.L., Boyne M.T., Yeh E., Vosburg D.A., Galonic D.P., Kelleher N.L., Walsh C.T. (2007). Characterization of the aminocarboxycyclopropane-forming enzyme CmaC. Biochemistry.

[11] Rangaswamy V., Jiralerspong S., Parry R., Bender C.L. (1998). Biosynthesis of the *Pseudomonas* polyketide coronafacic acid requires monofunctional and multifunctional polyketide synthase proteins. Proc. Natl. Acad. Sci. USA.

[12] Seidle H., Rangaswamy V., Couch R., Bender C.L., Parry R.J. (2004). Characterization of Cfa1, a monofunctional acyl carrier protein involved in the biosynthesis of the phytotoxin coronatine. J. Bacteriol..

[13] Rangaswamy V. (2000). ; Bender C.L. Phosphorylation of CorS and CorR, regulatory proteins that modulate production of the phytotoxin coronatine in *Pseudomonas syringae*. FEMS Microbiol. Lett..

[14] Peñaloza-Vázquez A., Bender C.L. (1998). Characterization of CorR, a transcriptional activator which is required for biosynthesis of the phytotoxin coronatine. J. Bacteriol..

[15] Wang L., Bender C.L., Ullrich M.S. (1999). The transcriptional activator CorR is involved in biosynthesis of the phytotoxin coronatine and binds to the *cmaABT* promoter region in a temperature-dependent manner. Mol. Gen. Genet..

[16] Alarcón-Chaidez F.J., Keith L., Zhao Y., Bender C.L. (2003). RpoN (σ^54^) is required for plasmid-encoded coronatine biosynthesis in *Pseudomonas syringae*. Plasmid.

[17] Smirnova A.V., Ullrich M.S. (2004). Topological and deletion analysis of CorS, a *Pseudomonas syringae* sensor kinase. Microbiology.

[18] Shu C.J., Ulrich L.E., Zhulin I.B. (2003). The NIT domain: a predicted nitrate-responsive module in bacterial sensory receptors. Trends Biochem. Sci..

[19] Gerharz T., Reinelt S., Kaspar S., Scapozza L., Bott M. (2003). Identification of basic amino acid residues important for citrate binding by the periplasmic receptor domain of the sensor kinase CitA. Biochemistry.

[20] Bender C.L., Liyanage H., Palmer D., Ullrich M., Young S., Mitchell R. (1993). Characterization of the genes controlling the biosynthesis of the polyketide phytotoxin coronatine including conjugation between coronafacic and coronamic acid. Gene.

[21] Keane P.J., Kerr A., New P.B. (1970). Crown gall of stone fruit. 2. Identification and nomenclature of *Agrobacterium* isolates. Aust. J. Biol. Sci..

[22] Weingart H., Stubner S., Schenk A., Ullrich M.S. (2004). Impact of temperature on in planta expression of genes involved in synthesis of the *Pseudomonas syringae* phytotoxin coronatine. Mol. Plant-Microbe Interact..

[23] Kovach M.E., Phillips R.W., Elzer P.H., Roop R.M., Peterson K.M. (1994). pBBR1MCS: a broad-host-range cloning vector. BioTechniques.

[24] Schenk A., Weingart H., Ullrich M.S. (2008). Extraction of high-quality bacterial RNA from infected leaf tissue for bacterial *in planta* gene expression analysis by multiplexed fluorescent Northern hybridization. Mol. Plant Pathol..

[25] Herrin D.L., Schmidt G.W. (1988). Rapid, reversible staining of northern blots prior to hybridization. BioTechniques.

[26] Bradford M.M. (1976). Rapid and sensitive method for quantitation of microgram quantities of protein utilizing principle of protein-dye binding. Anal. Biochem..

[27] Dunleavy J.M., Wylie T.D., Scott D.H. (1988). Bacterial, fungal, and viral diseases affecting soybean leaves. Soybean Diseases of the North Central Region.

[28] Smirnova A., Li H., Weingart H., Aufhammer S., Burse A., Finis K., Schenk A., Ullrich M. (2001). S. Thermoregulated expression of virulence factors in plant-associated bacteria. Arch. Microbiol..

[29] Graumann P., Marahiel M.A. (1996). Some like it cold: response of microorganisms to cold shock. Arch. Microbiol..

[30] Aguilar P.S., Lopez P., de Mendoza D. (1999). Transcriptional control of the low-temperature-inducible *des* gene, encoding the Δ5 desaturase of *Bacillus subtilis*. J. Bacteriol..

[31] Los D.A., Ray M.K., Murata N. (1997). Differences in the control of the temperature-dependent expression of four genes for desaturases in *Synechocystis* sp. PCC 6803. Mol. Microbiol..

[32] Thieringer H.A., Jones P.G., Inouye M. (1998). Cold shock and adaptation. BioEssays.

[33] Ullrich M.S., Schergaut M., Boch J., Ullrich B. (2000). Temperature-responsive genetic loci in the plant pathogen *Pseudomonas syringae* pv. glycinea. Microbiology.

[34] Andersen J.B., Sternberg C., Poulsen L.K., Bjørn S.P., Givskov M., Molin S. (1998). New unstable variants of green fluorescent protein for studies of transient gene expression in bacteria. Appl. Environ. Microbiol..

[35] Smirnova A.V., Wang L., Rohde B., Budde I., Weingart H., Ullrich M.S. (2002). Control of temperature-responsive synthesis of the phytotoxin coronatine in *Pseudomonas syringae* by the unconventional two-component system CorRPS. J. Mol. Microbiol. Biotechnol..

[36] Vaillancourt F.H., Yeh E., Vosburg D.A., O'Connor S.E., Walsh C.T. (2005). Cryptic chlorination by a non-haem iron enzyme during cyclopropyl amino acid biosynthesis. Nature.

